# Supplementary material for: The Effect of High and Low Ambient Temperature on Infant Health: A Systematic Review
Source: Int J Environ Res Public Health. 2022 Jul 26;19(15):9109. doi: 10.3390/ijerph19159109 (PMC9331681; doi:10.3390/ijerph19159109)
Supplement: Supplementary file 1 [file ijerph-19-09109-s001.zip › ijerph-1787584-supplementary.pdf]

Table S1. Search terms in Medline (PubMed).

Concept	Search Terms
Temperature (Exposure)	(ambient temperature* or ambient heat or ambient cold or heatwave* or heat wave* or heat event* or cold spell* or (extreme adj3 temperature*) or extreme heat or extreme cold or outdoor temperature* or environmental temperature* or indoor temperature or hot temperature* or cold temperature* or weather or (extreme adj3 weather) or climat* or meteorologic* or heat exposure or cold exposure or heat index or cold index or heat episode* or cold episode)
Infant (Population)	(infan* or neonat* or baby or babies or paediatric* or pediatric* or newborn*)
Adverse events (Outcome)	(adverse outcome* or mortalit* or death* or hospital admission* or hospital stay* or hospital visit or morbidit* or sudden infant death or SIDS or infect* or sepsis or emergenc* or hospitali#ation or asthma or cardiovascular or respiratory or hyperthermi* or hypothermi* or breastf?ed* or dehydration or hypoxia or anoxia or irritability or heat stress or cold stress or heat strain or heat exhaustion or heat stroke or heatstroke or overheating)

Table S2. Modified OHAT Risk of Bias Tool.

The sections italised and highlighted in blue text are the changes that were made to the original tool.

**Internal Validity**	**Question**	***Considerations***
Selection Bias	Did selection of study participants result in appropriate comparison groups?	Is the case definition adequate?<br>Is the control group comparable to the cases?
Confounding Bias	Did the study design/analysis account for important confounding and modifying variables?	Was confounding for seasonality controlled?<br>Was confounding for time trends, air pollution, other meteorological factors (rain, humidity), days of the week and holidays, influenza epidemics, sex (dependent on study design) controlled for?<br>Are the confounding variables measured validly and reliably?
Detection Bias	Can we be confident in the exposure characterisation?	Was the weather data obtained from an appropriate weather station?<br>Were the exposure variables appropriately aggregated spatio-temporally?<br>Were heat days accurately measured?<br>Were the comparison days chosen appropriately?<br>Was there a change in the measurement of exposure variables over time that could affect the results?<br>Was the effect of time lag of exposure on outcome taken into consideration?
	Can we be confident in the outcome assessment?	Was the outcome accurately measured to minimise information bias?<br>Were excess heat-related mortalities calculated accurately?<br>Was there a change in measurement of outcomes variables over time that could affect the results?<br>Were the exposure variables appropriately aggregated spatio-temporally?<br>Could the outcome measure have been influenced by knowledge of the exposure received?<br>Were the methods of outcome assessment comparable across exposure groups?
Selective reporting bias	Were all measured outcomes reported?	
Conflicts of interest	*Were there any conflicts of interest?*	
Analysis	*Were the statistical methods appropriate?*	Were any sensitivity analyses carried out?<br>**Was the study sufficiently powered to detect an intervention effect (if one exists)?**<br>Is a power calculation presented? If not, what is the expected effect size? Is the sample size adequate?
Other	Were there no other potential threats to internal validity?	Did the researchers adhere to the study protocol?
